# The diffuse interface description of fluid lipid membranes captures key features of the hemifusion pathway and lateral stress profile

**DOI:** 10.1093/pnasnexus/pgae300

**Published:** 2024-07-25

**Authors:** Matteo Bottacchiari, Mirko Gallo, Marco Bussoletti, Carlo M Casciola

**Affiliations:** Department of Basic and Applied Sciences for Engineering, Sapienza University of Rome, via Antonio Scarpa 16, Rome 00161, Italy; Department of Mechanical and Aerospace Engineering, Sapienza University of Rome, via Eudossiana 18, Rome 00184, Italy; Department of Mechanical and Aerospace Engineering, Sapienza University of Rome, via Eudossiana 18, Rome 00184, Italy; Department of Mechanical and Aerospace Engineering, Sapienza University of Rome, via Eudossiana 18, Rome 00184, Italy; Department of Mechanical and Aerospace Engineering, Sapienza University of Rome, via Eudossiana 18, Rome 00184, Italy

**Keywords:** vesicle fusion, vesicle fission, energy landscape, phase-field

## Abstract

Topological transitions of lipid membranes are ubiquitous in key biological processes for cell life, like neurotransmission, fertilization, morphogenesis, and viral infections. Despite this, they are not well understood due to their multiscale nature, which limits the use of molecular models and calls for a mesoscopic approach such as the celebrated Canham–Helfrich one. Unfortunately, such a model cannot handle topological transitions, hiding the crucial involved forces and the appearance of the experimentally observed hemifused intermediates. In this work, we describe the membrane as a diffuse interface preserving the Canham–Helfrich elasticity. We show that pivotal features of the hemifusion pathway are captured by this mesoscopic approach, e.g. a (meta)stable hemifusion state and the fusogenic behavior of negative monolayer spontaneous curvatures. The membrane lateral stress profile is calculated as a function of the elastic rigidities, yielding a coarse-grained version of molecular models findings. Insights into the fusogenic mechanism are reported and discussed.

**Significance Statement**

Fusion and fission of lipid membranes is a key step in countless biological processes and simultaneously involves large-scale membrane deformations and a local rearrangement of lipids. The need to follow both scales prevents the molecular simulation of these processes and thus the possibility of understanding the interaction between the two scales. Here, we show a method that contains both scales and use it to simulate vesicle fusion/fission with multiscale resolution. We also show how the elastic parameters governing the large-scale affect the distribution of stresses within the membrane, which is employed by cells to regulate certain membrane proteins such as mechanosensitive channels. Understanding how meso-/macroscopic observables influence the smaller scale is a fundamental step toward controlling such processes.

## Introduction

Widespread in key biological processes, from neurotransmission to fertilization, via morphogenesis and viral infections, topological transitions of fluid lipid membranes are an interdisciplinary research field of biological, biophysical, medical, pharmaceutical, and engineering interest (1–5).

A classical, elastic description of these membranes relies on the Canham–Helfrich model (6–8), which assigns a curvature-dependent energy to the lipid bilayer mid-surface. Denoting with *M* the mean curvature of such a surface, and with *G* the Gaussian curvature, the Canham–Helfrich energy reads


(1) $$E_{\mathrm{CH}}[\Gamma ]=2k\int_{\Gamma }(M-m{)}^{2}\;dS+k_{G}\int_{\Gamma }G\;dS,$$

where *Γ* represents the bilayer mid-surface and *m* is the so-called *spontaneous curvature*, which sets a preferred membrane curvature caused by some asymmetry between the two membrane leaflets. Hence, the energy has two contributions: a bending energy (first term on the right-hand side) with which a bending rigidity $k\approx 20\;k_{B}T$ (9) is associated, and a Gaussian energy contribution (second term) with an associated Gaussian modulus $k_{G}\approx -k$ (10). In particular, the Gaussian energy has a leading role during topological transitions due to the Gauss–Bonnet theorem of differential geometry, which states that the integral of *G* over a compact surface is a topological invariant, which for closed lipid vesicles yields


(2) $$\int_{\Gamma }G\;dS=4\pi (1-g),$$

where *g* is the genus of *Γ* and equals the number of holes in the surface, e.g. g=0 for a sphere and g=1 for a toroidal vesicle. Therefore, the Gaussian energy makes no contribution in the absence of topological changes, while providing large energy jumps and drops during fusion and fission events. These processes can happen via two distinct modes, referred to as *trans-* and *cis-*modes by Ishihara and coauthors (3), which are related to a change in the number of vesicles (ΔN) or to a change in the topological genus (Δg) of a single vesicle, respectively. For example, two spherical vesicles can merge into a single one (ΔN=−1) or, vice-versa, a spherical vesicle can be divided into two (ΔN=+1). These two opposite processes lead to a Gaussian energy variation of $4\pi k_{G}\Delta N$. On the other hand, a single vesicle can change its genus, e.g. a spherical vesicle can be pierced to obtain a torus (Δg=+1) or, vice-versa, a toroidal vesicle can rearrange into a spherical vesicle (Δg=−1), both processes with a Gaussian energy variation of $-4\pi k_{G}\Delta g$.

The description provided by the Canham–Helfrich model is essential since the full-scale evolution of large and giant vesicles is not currently achievable with molecular simulations (11, 12), which, however, provide valuable information on the local rearrangement of lipids that occurs during topological transitions, highlighting intermediate configurations (13, 14), their free energies (15–18), and the influence of microscopic details on them (19, 20). Unfortunately, the Canham–Helfrich approach cannot handle topological transitions since it treats the membrane as an infinitely thin (sharp) surface that thus cannot continuously change topology, requiring cuts to be introduced in the surfaces during fusion or fission events. Furthermore, the Canham–Helfrich energy, Eq. 1, is scale-invariant, whereas topological transitions are not, for which relative distances between approaching membrane segments matter and an additional microscopic scale given by the membrane thickness (∼5nm) should be considered. It is such a scale, at the level of which the topological rearrangement occurs, that confers a multiscale character to fusion and fission of large vesicles (size of 100 to 1000 nm). The sharp interface of the Canham–Helfrich approach cannot describe (semi-)merged intermediate states, which influence the large-scale path to fusion or fission. Accordingly, the Canham–Helfrich model allows evaluation of the crucial Gaussian contribution only an instant before and an instant after the merging process, hiding its significant associated forces in the missing gap. In order to overcome these issues, we have recently introduced a Ginzburg–Landau type of free energy that considers the bilayer as a diffuse interface (21), thus introducing an additional length scale related to the membrane thickness. In the limit of small interface width (sharp-interface limit), the Ginzburg–Landau free energy reproduces the Canham–Helfrich elasticity but has the additional ability to handle topological transitions in a natural and continuous way. This feature allows the unique opportunity to access the elastic force field which drives the topological transformation. While we introduced the method as a rational way to regularize the singularity of the process and smoothly match the solution before and after the merging event, enabling the continuity of the topology change, it was not clear whether and to what extent the approach is able to bridge the gap toward the molecular scales. Indeed, a critical question that is central in all diffuse interface descriptions is whether the diffused character of the interface only allows the regularization of the singularities arising from the mathematical abstraction of the sharp interface or contains additional physics pertaining to the granular structure of matter.

In this work, by explicitly considering both the two distinct modes of topology change, we show that the diffuse nature of the interface captures pivotal features of the so-called hemifusion pathway. We show that the interface contains more information than might be expected based only on its sharp-interface limit, yielding a mesoscopic description of topological transitions in fluid lipid membranes. We initially compute the minimal energy pathway (MEP) for the piercing of a spherical vesicle, namely the transition between an oblate large unilamellar vesicle (LUV) and a toroidal one. We show that, in this case, a large bending (*M*-associated) energy barrier must be overcome not only in the fission direction but also in the fusion one. Such an energy barrier is associated with large-scale membrane deformations and starts to build up continuously before the Gaussian energy variation, which is instead determined by the membrane local rearrangement that changes the topology. Peculiarly, we find that the topological barrier associated with the Gaussian energy in the fusion direction is partially screened by the bending energy variation and, therefore, by the concomitant large-scale membrane deformation, a fact that highlights the multiscale nature of topological transitions and thus the need for a mesoscopic approach. The computed MEP also brings out a hemifusion-like (meta)stable intermediate, as observed in many fusion experiments (22–25). This fact gives us the opportunity to investigate the effect of the monolayer spontaneous curvature as mapped into the Gaussian modulus. We find results in accordance with the known fusogenicity of lipids with negative monolayer spontaneous curvatures (26), another feature that is therefore captured by the diffuse nature of the interface. In order to corroborate our mesoscopic perspective, we calculate the lateral stress profile of the interface, obtaining a coarse-grained version of profiles found with molecular models. The lateral stress profile is often computed in molecular simulations in order to extract the elastic constants of the membrane, while here it is calculated for the first time as the ratio $k_{G}/k$ varies, providing elastic insights into the fusogenic mechanism. In order to discuss the dependency of the stability of the hemifusion intermediate on curvatures, we finally consider the transition between two distinct spheres and a single vesicle of spherical topology as their size varies. Comparison with molecular dynamics results suggests that the stability of the hemifusion intermediate is much related to elasticity, while its energy barrier to molecular details.

## Results and discussion

### MEP for the spherical-to-toroidal topology change

Phase-field models are well-established techniques for in silico studies of several interfacial phenomena (27–32). The adopted diffuse interface approach relies on a phase-field function ϕ(x) defined everywhere in the host space $\Omega \subseteq R^{3}$ and that can assume values between −1 and +1. The space region with ϕ=−1 identifies the outer environment of the vesicle, while the ϕ=+1 region is the inner environment. The diffuse interface is associated with the small transition layer between these two values, thus identifying the bilayer mid-surface *Γ* with the ϕ=0 level set. Such a description, as opposed to the sharp-interface model of Canham–Helfrich, does not require any cuts to be introduced into the membrane surface during fusion and fission events, allowing a natural, continuous handling of topological transitions. As extensively explained in our previous work (21), an integral-type functional E[ϕ] is associated with each phase-field configuration, with the integral done over the entire domain *Ω*. Such a Ginzburg–Landau type of free energy, whose expression is recalled in Section ‘Materials and methods’, also depends upon a parameter ϵ which controls the diffuse interface width. If *A* is the surface area of the vesicle taken into account, then $D_{\mathrm{ve}}=\sqrt{A/\pi }$ is its characteristic length. In our previous work (21), we have shown that in the sharp-interface limit ($\lambda =\epsilon /D_{\mathrm{ve}}\ll 1$) the Ginzburg–Landau free energy recovers the Canham–Helfrich one, $E[\phi ]∼E_{CH}$. More precisely, $E[\phi ]=E_{B}[\phi ]+E_{G}[\phi ]$, with $E_{B}[\phi ]$ recovering the bending energy, as also used in other works (33–36), and $E_{G}[\phi ]$ recovering the Gaussian energy, as introduced in our previous work (21). Thus, working within this limit to retain the Canham–Helfrich elasticity, here, we compute the MEP for the transition between a large oblate vesicle (spherical topology) and a Clifford torus, which is the ground state of the Canham–Helfrich energy with toroidal topology. Furthermore, we assume symmetric membranes, m=0. By definition, the MEP is a curve on the energy landscape that connects the oblate vesicle and the Clifford torus, which are two stable states. The curve is parameterized by α∈[0,1], that is at each *α* there is a vesicle configuration $\phi_{\alpha }$, with $\phi_{\alpha =0}$ that corresponds to the oblate vesicle and $\phi_{\alpha =1}$ to the Clifford torus. Denoting with $\delta E/\delta \phi_{\alpha }$ the functional derivative of E[ϕ] calculated at *α*, the MEP is such that $\partial \phi_{\alpha }/\partial \alpha \propto \delta E/\delta \phi_{\alpha }$, that it is everywhere tangent to the gradient of the potential, except at critical points where $\delta E/\delta \phi_{\alpha }\equiv 0$ (37). Here, the MEP is numerically found by means of the *string method* (38), a rare event technique that discretizes the pathway into a string of *N* images. Configurations along the path all share the same reduced volume $v=V/(\pi \;D_{\mathrm{ve}}^{3}/6)=0.71$, since it is assumed the conservation of the surface area *A* and enclosed volume *V* of the vesicle, see Section ‘Materials and methods’. The size of the vesicle is determined by the matching of the diffuse interface width with the bilayer thickness $6\epsilon =\ell_{me}=5\;\text{nm}$, Section ‘Materials and methods’. Hence, in the present case, vesicle configurations along the MEP are LUVs, with $D_{\mathrm{ve}}=211\;\text{nm}$ (λ=0.00395).

**Fig. 1.**
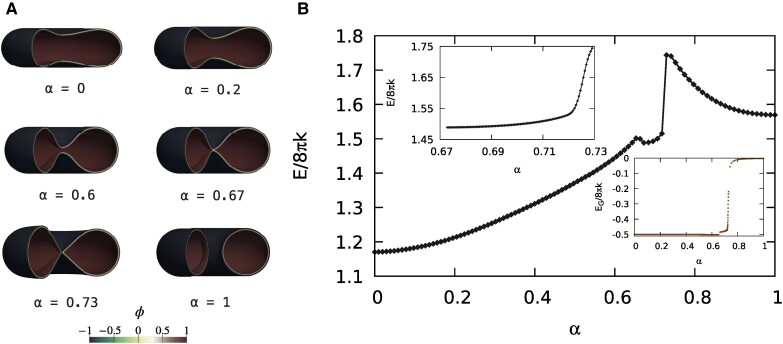
The MEP connecting an oblate vesicle to a toroidal one. All the configurations along the path share the same surface area and enclosed volume and therefore have fixed reduced volume v=0.71, as well as constant zero spontaneous curvature, m=0, and $k_{G}=-k$. The computation has been carried out with the string method, by assuming *z*-axial symmetry, in a [0ϵ,164ϵ]×[−75ϵ,75ϵ] computational domain in the *r–z* plane with a grid of 246×225 nodes per image of the string. All the configurations are LUVs, with $D_{\mathrm{ve}}=211\;\text{nm}$ (λ=0.00395). A) Six shapes along the MEP. In the forward direction (increasing the string parameter *α*), the oblate vesicle (α=0) starts to deform in order to be pierced. At α=0.67, an hemifusion-like configuration is achieved, which eventually evolves to the Clifford torus (α=1). In the backward direction, the division of the torus is apparent. B) The Ginzburg–Landau free energy E[ϕ] along the MEP, made up of N=100 images. There are three numerical minima at α=0, α=0.67, and α=1, and two maxima (saddle-points) at α=0.66 and α=0.73. The second one sets the energy barrier for the forward and backward processes. The main inset (top) depicts a refinement of the steepest stretch of the MEP, obtained with further 100 images. The second inset (bottom) shows the Gaussian energy contribution along the MEP ($E[\phi ]=E_{B}[\phi ]+E_{G}[\phi ]$, with $E_{B}$ the bending component and $E_{G}$ the Gaussian one). The energy jump prescribed by the Gauss–Bonnet theorem due to the topology change is apparent.

**Fig. 2.**
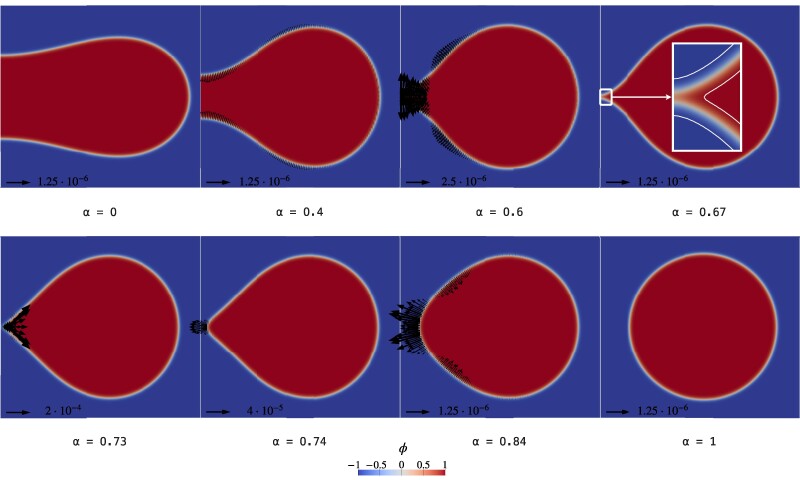
Detailed views in the *r–z* plane of the vesicle configurations, indexed with the string parameter *α*. Contours show the phase-field ϕ(x), while vectors provide the force field $f^{*}$ required to keep the vesicle in equilibrium in the given configuration, balancing the internal elastic reaction. In each plot, there is a dimensionless reference arrow. Indeed, the force fields are rescaled by using 8πk as the reference energy, and ϵ as the reference length. For the α=0.67 configuration, an enlargement of the merging region is also shown, in which the white lines are the $\phi =\text{tanh}(\pm 3/\sqrt{2})$ isolines that identify the beginning and end of the diffuse interface representing the membrane (21). The enlargement shows a hemifused arrangement of the interface in the merging region.

Figure 1A shows six different configurations along the MEP, each identified by its own string parameter *α*. Proceeding in the forward direction (increasing *α*), the cis-fusion of the vesicle is apparent. Indeed, the oblate vesicle (α=0) starts to deform in order to be pierced (α=0.2, α=0.6). At α=0.67 an hemifusion-like configuration is achieved, which deforms (α=0.73) and eventually evolves to the Clifford torus (α=1). The path traveled in the opposite (backward) direction corresponds to the cis-fission of the toroidal vesicle. Figure 1B (main plot) depicts the Ginzburg–Landau free energy E[ϕ] along the MEP. First of all, it is worth noticing that the oblate shape has $E[\phi_{\alpha =0}]/8\pi k=1.17$, that can be divided into a bending contribution $E_{B}[\phi_{\alpha =0}]/8\pi k=1.67$ and a Gaussian contribution $E_{G}[\phi_{\alpha =0}]/8\pi k=-0.5$, thus in accordance with the phase-diagram of Seifert and Lipowsky (39) for the Canham–Helfrich energy. Also the Clifford torus well captures the sharp-interface limit (40), with $E[\phi_{\alpha =1}]/8\pi k=1.57$ ($E_{B}[\phi_{\alpha =1}]/8\pi k=1.57$, $E_{G}[\phi_{\alpha =1}]/8\pi k=-2.30\cdot 10^{-3}$). Along the MEP, there are three numerical minima of the energy at α=0, α=0.67 and α=1, and two maxima (saddle-points) at α=0.66 and α=0.73. The second one sets the energy barrier for the forward and backward processes, $\Delta E_{0\to 1}^{†}=E[\phi_{\alpha =0.73}]-E[\phi_{\alpha =0}]$ and $\Delta E_{1\to 0}^{†}=E[\phi_{\alpha =0.73}]-E[\phi_{\alpha =1}]$, respectively. These barriers turn out to be $\Delta E_{0\to 1}^{†}/8\pi k\approx 0.57$ and $\Delta E_{1\to 0}^{†}/8\pi k\approx 0.17$, which are very high values, that prevent the processes from being thermally activated. Indeed, for $k=20\;k_{B}T$, they are $286\;k_{B}T$ and $85\;k_{B}T$, respectively. The string is made up of N=100 images, of which 70 are placed between α=0 and α=0.67 and are equidistant with respect to the norm induced by the standard $L^{2}$ inner product of the phase-field (21), while the remaining 30 are equally spaced in the remainder of the path. The main inset (top) of Fig. 1b depicts additional 100 images as a refinement of the steepest stretch of the MEP, obtained using the same procedure described in our previous work (21). This stretch of the MEP is placed between the aforementioned local minimum at α=0.67 and the saddle-point at α=0.73. As apparent from Fig. 1a, such a minimum consists of a hemifusion-like shape, which is therefore a (meta)stable configuration in the present case, as also observed in many experiments (22–25). This feature is not present in the pathway for the topological transition between two large spheres and one large sphere (21), where intermediates reminiscent of the stalk/hemifusion configurations were found to be unstable. The energy needed to escape from this (meta)stable stalk/hemifusion-like configuration in the forward direction is $\Delta E_{0.67\to 1}^{†}/8\pi k=0.256$. The small inset (bottom) of Fig. 1B shows the Gaussian energy variation along the MEP, which behaves as prescribed by the Gauss–Bonnet theorem, Eq. 2, thus remaining constant far from the topological transition. Therefore, in the present case, not only the backward barrier builds up continuously with the membrane deformation, but also the forward one, and is therefore associated to a bending energy variation. This characteristic was not present in the transition between two spheres and one sphere (21). Furthermore, the saddle-point is achieved when the Gaussian energy is still varying, thus when the merging process is not yet completed. This shows that, for the present system, the Gaussian energy jump that usually prevents fusion processes is screened by the bending energy, that is by the large-scale membrane relaxation. The underlying mechanics is more evident in Fig. 2, where detailed views in the *r–z* plane are provided. The contours show the phase-field $\phi_{\alpha }(x)$, while vectors provide the external force field f=−δE/δϕ∇ϕ needed to counterbalance the membrane elastic reaction in order to keep the vesicle in equilibrium in each configuration along the MEP. As explained in our previous work (21), these forces are those that can drive the transition under quasi-static conditions, therefore spending the minimal work. Of course, the displayed external forces drive the process in the forward direction until α=0.73, while drive the backward process from α=1 to α=0.73. The fact that vectors reverse their directions between α=0.73 and α=0.74 is a numerical confirmation that the saddle-point is actually located between these two images. It is worth saying that vectors are rescaled for each configuration in accordance with the provided reference arrow, which has a dimensionless magnitude obtained using the bending energy of a sphere (8πk) as the reference energy, and the diffuse interface width parameter ϵ as the reference length. Also shown in Fig. 2 is a close-up of the merging region at α=0.67, in which the white lines are the $\phi =\text{tanh}(\pm 3/\sqrt{2})$ isolines that identify the beginning and end of the membrane interface (21). The close-up shows a hemifused arrangement of the interface in the merging region (22), with the ϕ>0 part that is merged, while the ϕ<0 part is still separated.

Of course, several microscopic effects are not included in this diffuse interface approach but the energetic correction due to such microphysics should be small as compared to the elastic energy barriers computed here and associated with the full-scale evolution of the vesicle.

### Different Gaussian moduli

**Fig. 3.**
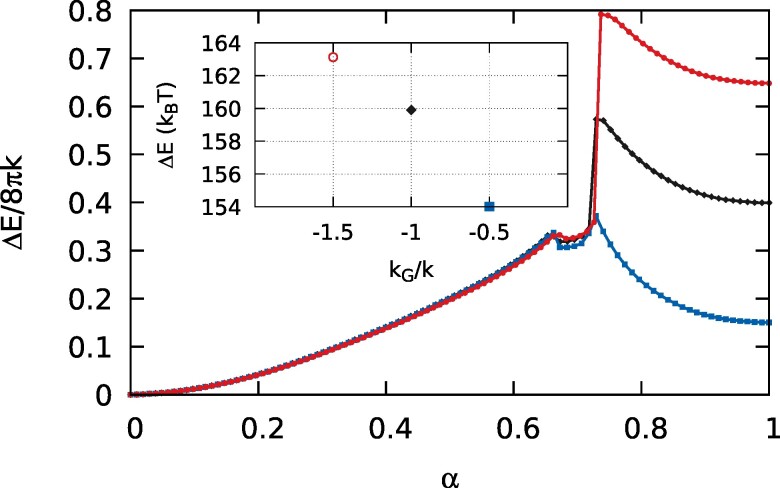
The Ginzburg–Landau free energy variation along the MEP for the transition between an oblate vesicle and a Clifford torus, with three different Gaussian moduli: $k_{G}/k=-0.5$ (line with squares, blue), $k_{G}/k=-1$ (line with diamonds, black), and $k_{G}/k=-1.5$ (line with circles, red). The three MEPs have been obtained with the string method, and the same parameters of Fig. 1, except for the Gaussian modulus. Actually, the case with $k_{G}/k=-1$ is exactly the same string of Fig. 1, whereas a refinement of the steepest stretch of the MEP with $k_{G}/k=-1.5$ has been carried out to confirm the result. The inset shows the Ginzburg–Landau excess free energies of the (meta)stable hemifusion-like intermediates found in the three cases, with $k=20\;k_{B}T$.

The fact that we found a (meta)stable stalk/hemifusion-like intermediate gives us the opportunity to study the effect that a different Gaussian modulus has on it. In fact, even if there is evidence that $k_{G}$ is roughly −k (10, 41), such a modification can probe the physics captured by the diffuse nature of the interface by virtue of the relationship between the Gaussian modulus and the monolayer spontaneous curvature $m^{ml}$,


(3) $$k_{G}=2\left(k_{G}^{\mathrm{ml}}-k^{\mathrm{ml}}z_{0}m^{\mathrm{ml}}\right),$$

where $z_{0}$ is a measure of the bilayer thickness, assumed to be symmetric, while $k^{\mathrm{ml}}$ and $k_{G}^{\mathrm{ml}}$ are the bending rigidity and Gaussian modulus (which is usually negative (42)) of the two constituent monolayers, respectively (43). Therefore, since it is known that lipids with a negative monolayer spontaneous curvature are more fusogenic (26), in the sense that their shapes favor the rearrangement in the stalk/hemifusion configuration, probing the behavior of the diffuse interface under a $k_{G}$ variation may bring out new insights. For this purpose, Fig. 3, main plot, shows the MEP for three different Gaussian moduli, $k_{G}/k=-0.5$ (line with squares, blue), $k_{G}/k=-1$ (line with diamonds, black), and $k_{G}/k=-1.5$ (line with circles, red). The system is always the same (oblate to Clifford torus), and the parameters are exactly the same of Fig. 1, except for the Gaussian modulus. First of all, a variation between the three cases in the forward energy barrier due to a rigid translation of the fission branch is apparent, with a much reduced value for $k_{G}/k=-0.5$, which corresponds to a more negative monolayer spontaneous curvature. This feature was already present in the transition between two large spherical vesicles and a single one (21). In addition to that, now, there is also an enhanced (meta)stability for the negative monolayer spontaneous curvature case, showing that the diffuse nature of the interface, together with its elasticity, is able to capture such a behavior as coarse-grained into the Gaussian modulus, the key elastic parameter for topological transitions of fluid lipid vesicles. Inset of Fig. 3 depicts the excess freeenergy (with respect to α=0) of the three (meta)stable hemifusion-like intermediates, calculated by assuming $k=20\;k_{B}T$.

### Lateral stress profile

**Fig. 4.**
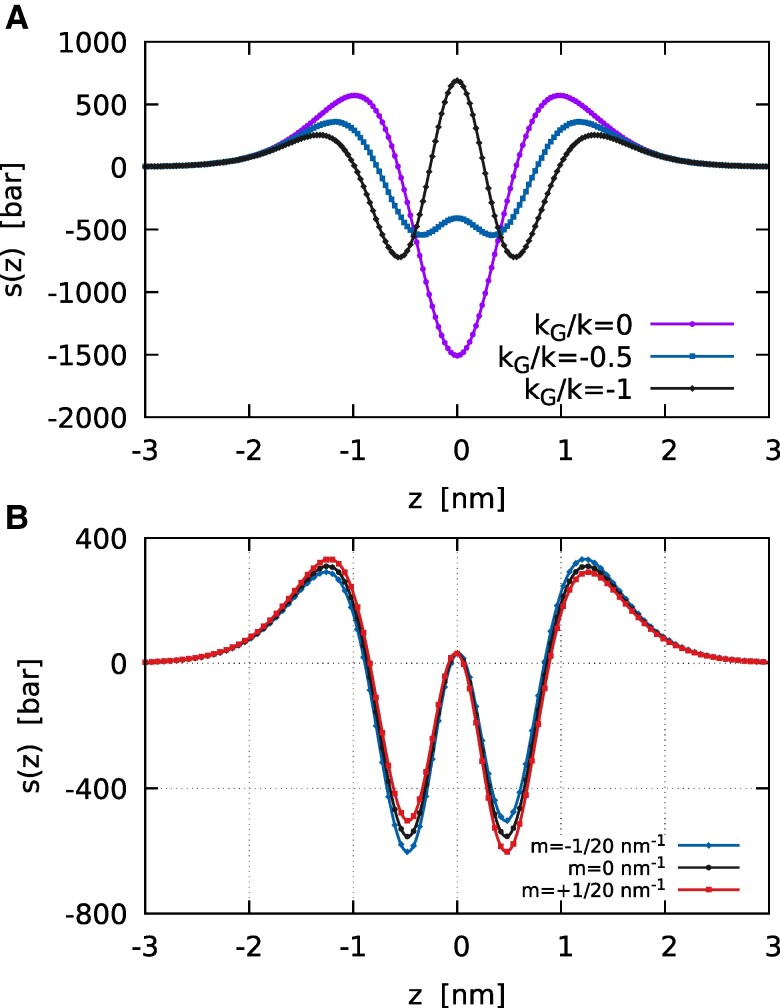
A) The lateral stress profile s(z) of a symmetric (m=0) diffuse interface membrane, with $k=20\;k_{B}T$. As $k_{G}/k$ varies different shapes are apparent. In particular, as $k_{G}$ decreases an interfacial tension between the two constituent monolayers at the center of the bilayer begins to appear. The case with $k_{G}/k=-2$ (not shown) has the same qualitative trend of that with $k_{G}/k=-1$, but with a much higher absolute maximum of about 2,890bar and the two minima of about −1,500bar. B) The lateral stress profile s(z) of a diffuse interface membrane, with $k=20\;k_{B}T$ and $k_{G}/k=-0.7$. Three cases are reported: the black line with circles shows s(z) for a symmetric membrane (m=0), while the other two curves depict the case of an asymmetric membrane with $m=-1/20\;{\text{nm}}^{-1}$ and $m=+1/20\;{\text{nm}}^{-1}$. Here, z<0 can be interpreted as the inner leaflet (ϕ>0), while z>0 as the outer leaflet (ϕ<0). It is worth noticing that the lateral stress of the two panels can be directly rescaled with *k* in order to consider different bending rigidities.

The mesoscopic point of view of the here adopted Ginzburg–Landau phase-field method allows the large-scale simulation of topological transitions of lipid vesicles. On the one hand, the approach is convenient since it enhances the celebrated Canham–Helfrich model with the ability to handle topology changes, thus providing access to spatio-temporal scales unattainable by molecular models (11). On the other hand, as already pointed out, the Ginzburg–Landau model lacks in molecular details. For this reason, the results presented so far on the hemifusion intermediate are somewhat surprising and suggest that the diffuse nature of the interface considered here adds much to the elastic description of membranes, more than one might simply think on the basis of the sharp-interface limit. Therefore, in an attempt to understand what connections there are with the molecular scales, we now calculate the lateral stress profile of the diffuse interface and interpret it by considering the presence of two hypothetical lipid leaflets on the two sides of the interface mid-plane ϕ=0, that is a leaflet on the ϕ<0 side and the other one on the ϕ>0 side. The lateral stress profile is often computed in molecular simulations since its associated moments are related to the elastic parameters of the membrane. Its determination is in general not obvious (44, 45), leads to odd results as regards the Gaussian modulus (41), and its distribution depends on the specific model taken into account. For example, the MARTINI model (41) shows repulsion between lipid heads, then an interfacial tension (attraction) at the hydrophilic/hydrophobic interface of several hundreds of bars, and a repulsive region due to the compression of lipid tails. Self-consistent field theory also reproduces the same qualitative behavior (46), with the addition of an interface tension between the opposing tails of the two constituent monolayers. The profile obtained with Dissipative Particle Dynamics (47) has an attractive head group region, with a double-peak corresponding to the water/lipid head and lipid head/chain interfaces, while the hydrocarbon tail region is still repulsive. The coarse-grained, implicit-solvent Cooke model (10, 48) provides yet another profile with a positive central peak. Unlike in these models, our lateral stress is provided as a function of the elastic coefficients *k* and $k_{G}$. Gompper and Zschocke (GZ) (49) have calculated the expression for the lateral stress profile in the context of a Ginzburg–Landau free energy functional. As illustrated in Section ‘Materials and methods’, the bending component $E_{B}$ of the here considered free energy can be rewritten in the form of GZ (49), with $c=3k\epsilon /(4\sqrt{2})$, $g(\phi )=3k(3\phi^{2}-1+2\sqrt{2}m\epsilon \phi )/(2\sqrt{2}\epsilon )$, $f(\phi )=3k(\phi^{2}-1{)}^{2}(\phi +\sqrt{2}\epsilon m{)}^{2}/(4\sqrt{2}\epsilon^{3})$, see also Lázaro et al. (50) for a review. Therefore, the calculations of GZ give us the lateral stress profile associated with the bending energy term, $s_{b}(z)$, which for symmetric membranes reads $s_{b}(z)=2g(\phi_{0})\phi_{0}^{\prime 2}/\epsilon^{2}+4c\phi_{0}^{\prime \prime 2}/\epsilon^{4}$, where $\phi_{0}=\phi_{0}(z/\epsilon )=\text{tanh}(-z/(\epsilon \sqrt{2}))$ is the planar solution of our Ginzburg–Landau free energy, *z* is the coordinate normal to the plane, and the prime denotes the derivative with respect to z/ϵ. The Gaussian contribution to the lateral stress profile is $s_{G}(z)=35k_{G}(12\phi_{0}^{\prime 2}\phi_{0}^{\prime \prime 2}+4\phi_{0}^{\prime 3}\phi_{0}^{\prime \prime \prime })/(16\sqrt{2}\epsilon^{3})$, and is derived in Section ‘Materials and methods’ by considering a spherical vesicle. The sum of these two contributions is the lateral stress profile of the membrane diffuse interface, $s(z)=s_{b}(z)+s_{G}(z)$, whose moments (λ≪1) provide


(4a) $$\int_{-\infty }^{+\infty }\;s(z)\;dz=\hat{\Sigma },$$

(4b) $$\int_{-\infty }^{+\infty }\;zs(z)\;dz=-2\;km,$$

(4c) $$\int_{-\infty }^{+\infty }\;z^{2}s(z)\;dz=k_{G}+2\;k,$$

being $\hat{\Sigma }=2\;km^{2}$ the spontaneous tension of the membrane. These results are in accordance with the calculations of GZ, see also Oversteegen and Leermakers (51) for a detailed discussion. Figure 4a depicts the lateral stress s(z) for a symmetric membrane, m=0, as $k_{G}/k$ varies—for the stability of the bilayer it must be $-2\leq k_{G}/k\leq 0$. When $k_{G}=0$ the profile includes two contributions (50). The profile is attractive (positive) in the regions of the head groups, which therefore tend to minimize the contact area with the surrounding aqueous environments, while it is repulsive (negative) in the lipid tail regions. As $k_{G}/k$ decreases, an interface tension between the opposing tails of the two constituent monolayers begins to appear, creating a stress bump at the bilayer mid-plane, which eventually becomes positive leading to a third region with an interfacial tension between the two monolayers. The case with $k_{G}/k=-1$ (black line with diamonds in Fig. 4A) actually has a positive central peak and looks very much like the profile calculated for a spherical vesicle by Oversteegen and Leermakers with a mean field lattice model—note that also here the derivation is based on a spherical configuration, Section ‘Materials and methods’. In our previous work (21), we showed that the Gaussian energy term provides a force field whose differential between the two leaflets tends to prevent membrane lysis, namely the separation of the two leaflets, which would amount to the expensive enlargement of the interface between the two monolayers. Interestingly, in the literature, the free volume available between the two leaflets is also associated with the accumulation of oxygen within the bilayer, which has important biological implications (52, 53). The central peak equals zero when $k_{G}/k=-24/35$. Incidentally, $k_{G}/k=-0.7$ is the value found by Hu et al. (41) with the MARTINI model if one considers an updated value of the bending rigidity (54). In this case ($k_{G}/k=-0.7$), the central peak is mildly positive as depicted in Fig. 4B, black line with circles. Positive central peaks that are small compared with the lateral ones are also found in atomistic simulations (44, 55).

On the basis of Eq. 3, the external induction of a negative monolayer spontaneous curvature, $m^{ml}<0$, leads to an increase in $k_{G}$ (less negative), which in turn leads to a more fusogenic bilayer. This induction is generated, for example, by molecules that preferentially insert into the hydrocarbon chain region, thereby increasing the compression of lipid tails, which in turn become more repulsive, see Koller and Lohner (56). This should indeed correspond to a reduction in the central stress peak, in accordance with the behavior shown in Fig. 4A. On the contrary, a molecule preferentially inserting in the head group region tends to increase $m^{ml}$ (56), leading to a reduction of $k_{G}$ (more negative, less fusogenic bilayer). In this case, the molecule compresses lipid heads and a mitigation in their corresponding positive peaks is indeed present in Fig. 4A. This reduction is balanced by an increase in the central stress bump.

Finally, Fig. 4B depicts the case with nonzero bilayer spontaneous curvature, where the peaks are not symmetric, reflecting the asymmetry between the two lipid leaflets. A nonzero bilayer spontaneous curvature can also be induced by the membrane adsorption of small solute (57) or by low densities of membrane bound proteins (58). It is worth saying that our lateral stress has peaks on the order of hundreds of bars as found in molecular models.

### Discussion

**Fig. 5.**
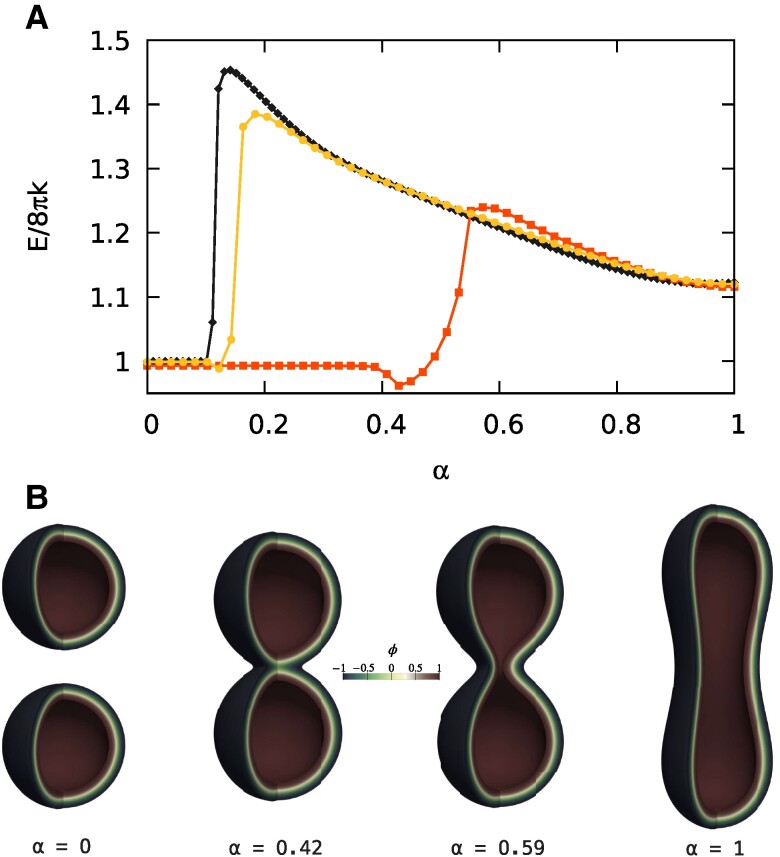
(A) The MEPs for the trans-type topological transition between two spherical vesicles and a dumbbell-shaped one, for three different vesicle sizes: $D_{\mathrm{ve}}\approx 206\;\text{nm}$ (line with diamonds, black), $D_{\mathrm{ve}}\approx 113\;\text{nm}$ (line with circles, yellow), and $D_{\mathrm{ve}}\approx 47\;\text{nm}$ (line with squares, orange). The larger case is reproduced from our previous work (21), and all the cases share the same elastic parameters ($k_{G}=-k$, m=0, $v=1/\sqrt{2}$) and preserve surface area and enclosed volume along the evolution. For the $D_{\mathrm{ve}}\approx 113\;\text{nm}$ case, we have used a [0ϵ,60ϵ]×[−135ϵ,135ϵ] computational domain in the *r–z* plane with a grid of 90×405 nodes per image of the string, N=50 images, λ=0.007. For the $D_{\mathrm{ve}}\approx 47\;\text{nm}$ case, we have used a [0ϵ,28ϵ]×[−60ϵ,60ϵ] computational domain in the *r–z* plane with a grid of 42×180 nodes per image of the string, N=50 images, λ=0.018. (B) Four vesicle configurations along the MEP, $D_{\mathrm{ve}}\approx 47\;\text{nm}$. Proceeding in the forward direction, at α=0 the two SUVs are distant from each other, then get in close apposition and start to merge, reaching an intermediate hemifusion-like stable state at α=0.42. The topological transition is accomplished at α=0.59, and the vesicle can eventually evolve to its final prolate shape α=1.

In this work, we have shown that a diffuse interface description of a fluid lipid membrane is not only able to allow access to topological transitions and the involved large-scale elastic forces, but it is capable of reproducing features related to the local behavior of the merging region. Intermediates reminiscent of those found in experiments (22–25), and molecular dynamics simulations (17, 18, 59) were already apparent in the transition between two large spherical vesicles and a dumbbell-shaped one (21). Here, we have additionally shown that the hemifusion-like intermediate can also be (meta)stable, and, furthermore, that its stability is enhanced by a negative monolayer spontaneous curvature, Eq. 3, and reduced by a positive monolayer spontaneous curvature, in accordance with known results (26). Moreover, an enhanced stability is also associated to a reduction in the fusion energy barrier, and, vice-versa, a reduced stability is matched with an increased barrier. Of course, the first reason one may think of for the stabilization of the hemifusion-like intermediate is curvature, due to the different vesicle shapes considered here with respect to the two spheres case. Therefore, in order to discuss our results, we push the model toward its limit with respect to the sharp-interface convergence, and consider the transition between two spheres and a dumbbell-shape for three different curvatures. In this regard, Fig. 5A shows the MEP for such a transition with $D_{\mathrm{ve}}\approx 206\;\text{nm}$ (line with diamonds, black), $D_{\mathrm{ve}}\approx 113\;\text{nm}$ (line with circles, yellow), and $D_{\mathrm{ve}}\approx 47\;\text{nm}$ (line with squares, orange). The larger case is reproduced from our previous work (21) and is extensively discussed there. All the cases share the same elastic parameters ($k_{G}=-k$, m=0, $v=1/\sqrt{2}$) and preserve surface area and enclosed volume along their own paths. As apparent, two additional stable configurations emerge in the $D_{\mathrm{ve}}\approx 113\;\text{nm}$ and $D_{\mathrm{ve}}\approx 47\;\text{nm}$ cases. As shown for the SUV case in Fig. 5B, these two minima correspond to stable hemifusion-like intermediates. Therefore, stability is enhanced with small radii and lost at large sizes. A closer inspection of the obtained configurations shows that in the three cases the distance at which the vesicles are brought before a local deformation starts the merging process is different. In particular, we find that the $\phi =\text{tanh}(-3/\sqrt{2})$ level sets that define the external end of the interfaces (21) are 1.16nm distant from each other in the last image of the neutral plateau region in the $D_{\mathrm{ve}}\approx 113\;\text{nm}$ case. Such a distance is reduced to 0.68nm in the SUVs case ($D_{\mathrm{ve}}\approx 47\;\text{nm}$), whereas for the larger case ($D_{\mathrm{ve}}\approx 206\;\text{nm}$) we found 3.89nm (21). Of course, these numbers should not be taken too seriously, because, for example, they depend on the choice of the level set that defines the end of the interface. Anyway, they bring to light the fact that the stability is enhanced decreasing the distance at which the vesicles are brought before deforming to start the merging process, a fact in accordance with the molecular dynamics results of Smirnova et al. (19) and Poojari et al. (20), which have shown that the initial distance at which the merging process begins is the most important factor for determining the energy of the stalk configuration (the closer the better).

As already discussed in our previous work (21), the plateau region of the energy in Fig. 5A corresponds to rigid translations of the two approaching spheres. This stretch of the MEP is energetically neutral due to the lack in molecular detail of the model, which, for example, does not consider the hydration repulsion barrier that must be overcome in this stage of the process, and that can be estimated analytically (59). Although this barrier is not even considered in the two previously mentioned molecular dynamics works (19, 20), the absence of molecular details in our model can be used in our favor so as to disentangle the elastic effects from the microphysics. In fact, in our case, Fig. 5A, no energy barrier is detected to reach the stalk/hemifusion-like configuration in the forward direction, while Smirnova et al. found a $20\;k_{B}T$ barrier independent of the initial distance between the merging membranes. This seems to suggest that the stability of the stalk/hemifusion configuration is much related to elasticity, while its associated energy barrier to molecular details, e.g. to the cost of exposing the hydrophobic tails of lipids to the aqueous environment. Indeed, by dividing $20\;k_{B}T$ by the oil–water interface tension, one is left with a surface area that can accommodate a small group of lipids that may form the initial stalk. Nonetheless, the lateral stress profile we found shows that our phase-field not only inherits the Canham–Helfrich elasticity of the membrane mid-surface but also provides a coarse-grained, mesoscopic picture of the bilayer through the diffuse interface. This addition led to results in accordance with experimental and numerical findings on the hemifusion pathway and is compatible with the elastic derivation of the Ginzburg–Landau free energy since the forces underlying membrane elasticity are indeed related to the amphiphilic nature of lipids. Although the model captures the main features of the lateral stress profile, further effects may be introduced through the addition of other elastic constants in the free energy, such as a nonlocal bending rigidity (60), a case we leave for a future work.

As a conclusion, let us discuss the SUV case in more detail. Figure 5A shows a reduced fusion energy barrier in the forward direction. In this case the model is pushed toward its limit with respect to the sharp-interface convergence and thus the result should be viewed with caution. Despite this, λ=0.018 seems to be small enough to reach convergence to the sharp-interface model of Canham–Helfrich, as apparent from the calculated energies at α=0 and α=1. Furthermore, the Gaussian component is still found to behave as prescribed by the Gauss–Bonnet theorem. Therefore, on the one hand, the energy barrier reduction seems to be due to the breaking of the scale invariance during topological transitions (relative distances matter). Indeed, the prolate shapes apparent at the end of the merging process in the SUV case resemble those found at similar *α* in the LUV case, as also a correspondence in their energies suggests (scale invariance holds after the merging process). On the other hand, the Gaussian energy is still varying partially after the saddle-point in the SUV case, indicating that the rearrangement of the interface is not yet fully completed. Nevertheless, the fusion barrier is still very large, in accordance with the stability and barrier function of cells. Therefore, Nature must have sophisticated mechanisms to lower it in order to allow biologically significant processes in a sufficiently fast way, e.g. by locally modifying the Gaussian modulus (61), see also Deserno (62). Anyway, if the process must be ultrafast as in neurotransmission (hundreds of microseconds), it is better to use small vesicles, Fig. 5A, and, furthermore, not only the elastic barrier must be lowered, but also those associated with the molecular detail. In this regard, Smirnova et al. (19) found that isolated transmembrane domains of the SNARE machinery indeed lower the $20\;k_{B}T$ stalk barrier. Incidentally, synaptic vesicles are small, whereas enveloped viruses can be large and lead to infections in several minutes.

## Materials and methods

### Ginzburg–Landau free energy for membranes

The here adopted diffuse interface approach relies on a phase-field function ϕ(x) defined everywhere in the host space $\Omega \subseteq R^{3}$ and that can assume values between −1 and +1. The space region with ϕ=−1 identifies the outer environment of the vesicle, while the ϕ=+1 region is the inner environment. The associated Ginzburg–Landau free energy reads


(5) $$E[\phi ]=E_{B}[\phi ]+E_{G}[\phi ],$$

where


(6) $$E_{B}[\phi ]=k\;\frac{3}{4\sqrt{2}}\;\epsilon \;\int_{\Omega }\Psi_{B}^{2}\;dV,$$

(7) $$\Psi_{B}=\nabla^{2}\phi -\frac{1}{\epsilon^{2}}(\phi^{2}-1)(\phi +\sqrt{2}\epsilon m),$$

and


(8) $$E_{G}[\phi ]=k_{G}\frac{35}{16\sqrt{2}}\epsilon^{3}\int_{\Omega }\Psi_{G}\;dV,$$

(9) $$\Psi_{G}=\frac{\nabla |\nabla \phi {|}^{2}\cdot \nabla |\nabla \phi {|}^{2}}{2}-(\nabla |\nabla \phi {|}^{2}\cdot \nabla \phi )\nabla^{2}\phi$$

(10) $$\;+|\nabla \phi {|}^{2}\left[(\nabla^{2}\phi {)}^{2}+\nabla \phi \cdot \nabla \nabla^{2}\phi -\frac{\nabla^{2}|\nabla \phi {|}^{2}}{2}\right].$$

$E_{B}[\phi ]$ models the bending energy of the membrane (33, 36, 50), while $E_{G}[\phi ]$ is the Gaussian term introduced in our previous work (21), where we have also shown that E[ϕ] recovers the Canham–Helfrich energy, $E[\phi ]∼E_{\mathrm{CH}}[\Gamma ]$, in the sharp-interface limit ($\lambda =\epsilon /D_{\mathrm{ve}}\ll 1$). Here, ϵ is a small parameter that controls the diffuse interface width and that is matched to the bilayer thickness, $6\epsilon =\ell_{me}=5\;\text{nm}$. This relation sets the scale of our simulations and is needed because the scale invariance is broken during topological transitions (relative distances between approaching membrane segments matter) (21).

The large tension associated with the area change does not allow membrane bending to significantly modify *A*. Furthermore, the enclosed volume *V* is often determined by the osmotic conditions. Hence, in order to conserve these two quantities along the MEP, we use suitable functionals A[ϕ] and V[ϕ] which recover the vesicle area and volume, respectively, in the sharp-interface limit:


(11) $$A[\phi ]=\frac{3}{4\sqrt{2}}\epsilon \int_{\Omega }\left[\frac{(1-\phi^{2}{)}^{2}}{2\epsilon^{2}}+|\nabla \phi {|}^{2}\right]\;dV,$$

(12) $$V[\phi ]=\int_{\Omega }\frac{\left(1+\phi \right)}{2}\;dV.$$

The MEP is obtained by means of the string method (38), with constraints imposed by an augmented Lagrangian method (21, 63).

### Lateral stress profile calculation

By comparing the Canham–Helfrich free energy of a cylindrical and a spherical vesicle with those of a Ginzburg–Landau free energy functional, GZ (49) have calculated the expression for the lateral stress profile in the Ginzburg–Landau context. By means of an integration by parts of the linear term in the Laplacian, the bending component $E_{B}$ of the here considered free energy, Eq. 6, can be rewritten as


(13) $$E_{B}[\phi ]\;=\int_{\Omega }\left[c(\nabla^{2}\phi {)}^{2}+g(\phi )|\nabla \phi {|}^{2}+f(\phi )\right]\;dV,$$

with $c=3k\epsilon /(4\sqrt{2})$, $g(\phi )=3k(3\phi^{2}-1+2\sqrt{2}m\epsilon \phi )/(2\sqrt{2}\epsilon )$, $f(\phi )=3k(\phi^{2}-1{)}^{2}(\phi +\sqrt{2}\epsilon m{)}^{2}/(4\sqrt{2}\epsilon^{3})$, which is the form of GZ (49), see also (50). Therefore, the calculations of GZ, which we do not repeat here, give us the lateral stress profile associated with the bending energy term, $s_{b}(z)$, which for symmetric membranes reads $s_{b}(z)=2g(\phi_{0})\phi_{0}^{\prime 2}/\epsilon^{2}+4c\phi_{0}^{\prime \prime 2}/\epsilon^{4}$, where $\phi_{0}=\phi_{0}(z/\epsilon )=\text{tanh}(-z/(\epsilon \sqrt{2}))$ is the planar solution of our Ginzburg–Landau free energy, *z* is the coordinate normal to the plane, and the prime denotes the derivative with respect to z/ϵ.

In our previous work (21), we have shown that the phase-field that minimizes the Ginzburg-Landau free energy $E=E_{B}+E_{G}$ has the form $\phi (x)=f_{0}(d^{*}(x))+O(\lambda^{2})$, with $f_{0}(d^{*}(x))=\text{tanh}(d(x)/\left(\epsilon \sqrt{2}\right))$, where d(x) is the signed distance function from the ϕ=0 level set that represents the bilayer mid-surface, while $d^{*}(x)=d(x)/\epsilon$. Denoting with a prime the derivative done with respect to $d^{*}(x)$, we have also shown with a direct computation that


(14) $$E_{G}[\phi ]=k_{G}\frac{35}{8\sqrt{2}}\int_{\bar{\Omega }}\frac{\;f_{0}^{\prime 4}}{\lambda }\bar{G}\;d\bar{V}+O(\lambda^{2}),$$

where the bar indicates that the lengths have been made dimensionless by dividing by $D_{\mathrm{ve}}$, and G(x) must be interpreted as the Gaussian curvature of the *ϕ*-level set passing through x. For a large ($\lambda =\epsilon /D_{\mathrm{ve}}\ll 1$) spherical vesicle—for which in spherical coordinates $d^{*}(r)=(D_{\mathrm{ve}}/2-r)/\epsilon =(1/2-\bar{r})/\lambda$—the last equality leads to


$$\begin{array}{rl} E_{G}[\phi ] & =k_{G}\frac{35}{8\sqrt{2}}4\pi \int_{0}^{+\infty }\frac{\;f_{0}^{\prime 4}((1/2-\bar{r})/\lambda )}{\lambda }\;d\bar{r}+O(\lambda^{2})\\ & =k_{G}\frac{35}{8\sqrt{2}}4\pi \int_{-\infty }^{1/\left(2\lambda \right)}f_{0}^{\prime 4}(z^{*})\;dz^{*}+O(\lambda^{2})\\ & \approx k_{G}\frac{35}{8\sqrt{2}}4\pi \int_{-\infty }^{+\infty }f_{0}^{\prime 4}(z^{*})\;dz^{*}\\ & =k_{G}\frac{35}{8\sqrt{2}}4\pi \int_{-\infty }^{+\infty }\frac{z^{*2}}{2}\left[12f_{0}^{\prime 2}f_{0}^{\prime \prime 2}+4f_{0}^{\prime 3}f_{0}^{\prime \prime \prime }\right]\;dz^{*}\\ & =4\pi \int_{-\infty }^{+\infty }z^{2}\;s_{G}(z)\;dz, \end{array}$$

where in the second-to-last step the dependencies on $z^{*}$ have been omitted, and $s_{G}(z)=35k_{G}(12\phi_{0}^{\prime 2}\phi_{0}^{\prime \prime 2}+4\phi_{0}^{\prime 3}\phi_{0}^{\prime \prime \prime })/(16\sqrt{2}\epsilon^{3})$—note that $\phi_{0}(z/\epsilon )=f_{0}(-z/\epsilon )=-f_{0}(z/\epsilon )$. The Canham–Helfrich Gaussian energy of a sphere is $4\pi k_{G}$ and, indeed, the second moment of $s_{G}(z)$ exactly equals $k_{G}$. Furthermore, both the zeroth and the first moments of $s_{G}(z)$ are zero. Therefore, $s_{G}(z)$ can be interpreted as the Gaussian contribution to the lateral stress and added to $s_{b}(z)$ to provide the lateral stress profile of the membrane diffuse interface, $s(z)=s_{b}(z)+s_{G}(z)$. The zeroth, first, and second moments of s(z), Eq. 4, exactly equal combinations of the elastic constants as previously reported in the literature (49, 51).

## Data Availability

The dataset of the simulations, the post-processed data used for the figures and the scripts that generate them have been deposited in the Zenodo database at https://doi.org/10.5281/zenodo.10666170.
